# Development and external validation of a risk prediction tool for recurrent wheeze attacks in preschool children in England

**DOI:** 10.1183/23120541.00337-2026

**Published:** 2026-10-19

**Authors:** Jonathan Broomfield, Erol A. Gaillard, Jennifer K. Quint, Hilary Pinnock, Clare L. Gillies, Mohammad A. Al Sallakh, Gwyneth A. Davies, David K.H. Lo

**Affiliations:** 1 Division of Respiratory Sciences, University of Leicester, Leicester, UK; 2 Division of Public Health and Epidemiology, University of Leicester, Leicester, UK; 3 School of Public Health, Imperial College London, London, UK; 4 Usher Institute, The University of Edinburgh, Edinburgh, UK; 5 Leicester Real World Evidence Unit, University of Leicester, Leicester, UK; 6 Population Data Science, Faculty of Medicine, Health and Life Science, Swansea University, Swansea, UK; 7 Paediatric Respiratory Medicine, University Hospitals of Leicester, Leicester, UK

## Abstract

**Introduction:**

Almost half of all children experience wheeze before their sixth birthday, with a subset experiencing recurrent attacks that increase healthcare burden. Early identification of children at high risk could enable timely interventions, but diagnostic tools are limited. The aim of the present study was to develop and validate a prognostic model predicting the risk of recurrent wheeze attacks (primary outcome) and hospitalisations (secondary outcome) within 12 months of an initial preschool acute wheeze presentation.

**Methods:**

We developed a multivariable logistic regression model using primary care data from 42 820 children in England aged up to 5 years with acute wheeze episodes recorded in the Clinical Practice Research Datalink. Predictors included demographics, clinical history and treatment patterns. The model was validated internally *via* bootstrapping and externally using the Secure Anonymised Information Linkage Databank in Wales (9703 children). Model performance was assessed using calibration and discrimination metrics.

**Results:**

In the CPRD development cohort, 39.6% experienced a subsequent wheeze attack and 17.4% were hospitalised. Key predictors were the healthcare setting of initial acute wheeze, prior wheeze history and reliever prescriptions; inhaled corticosteroid prescriptions reduced risk. The model demonstrated excellent calibration across internal bootstrap validation and two external validation cohorts (calibration slope=1.00, 0.94 and 0.86, respectively, calibration-in-the-large=0.00, 0.035 and −0.143) and moderate discrimination (C-statistic=0.634, 0.625 and 0.638 for wheeze).

**Conclusions:**

Using routinely collected data, the prognostic model shows strong calibration and moderate discrimination for predicting recurrent wheeze in preschool children. Implementation as a web tool could support early risk stratification and guide preventative management.

## Shareable abstract

**A validated prediction model using routine primary healthcare data can identify preschool children at low, medium or high risk of recurrent wheeze, supporting earlier targeted intervention and improving clinical management of future asthma diagnosis** https://bit.ly/4ucwcRZ

## Background

Wheezing illnesses are prevalent in preschool-aged children, with half experiencing at least one episode by 6 years [1–3]. While most children experience mild, infrequent episodes triggered by viral respiratory infections, a subset suffer recurrent or persistent symptoms between infections [4, 5]. These children often require frequent healthcare visits and endure a reduced quality of life. Importantly, they are more likely to be diagnosed with asthma later in childhood [6], and may benefit from earlier asthma treatment with regular inhaled corticosteroids (ICS) [7, 8].

In preschool children, confirming an asthma diagnosis remains challenging due to the impracticality of objective testing [9], contributing to clinical uncertainty around initiating long-term preventer therapy. Current guidelines recommend trialling ICS in children with frequent or severe wheezing, based primarily on clinical history rather than diagnostic confirmation [10, 11]. Despite rising rates of unscheduled healthcare attendances for acute wheeze, routine data show no corresponding increase in ICS prescriptions. Instead, there is growing reliance on short-acting β_2_-agonists (SABAs) and oral corticosteroids [6], reflecting a reactive, not preventative, approach to asthma management [12, 13].

Identifying children at risk of future wheeze attacks is a critical component of asthma care [10, 11]. However, published clinical prediction models for childhood asthma broadly focus on asthma diagnosis rather than wheeze attacks [14, 15]. Our previous research identified potential clinical risk and patient demographic factors associated with further attacks of wheeze in preschool children who have consulted acutely with wheeze in primary or secondary care [16]. This study aimed to extend this work to develop and validate a prognostic risk prediction model to identify children at increased risk of subsequent wheeze. Such a model could support targeted intervention and improve long-term outcomes in this population. The model was developed on children in England from the Clinical Practice Research Datalink (CPRD) Aurum database [17] and validated in two cohorts of children in Wales from the Secure Anonymised Information Linkage (SAIL) Databank [18–20].

The article is reported in line with the TRIPOD statement [21]; a checklist is available in the supplementary material.

## Methods

### Study population

The risk model development cohort comprised children aged 5 years and under registered with general practices contributing data to CPRD Aurum [17] with linkage available to Hospital Episode Statistics (HES) Admitted Patient Care [22], Outpatient [23] and Accident and Emergency [24] databases. Practices eligible for HES linkage (∼75% of CPRD practices in England) are representative of nonlinked practices [25].

Children ≤5 years presenting with at least one episode of acute wheeze between 1 January 2013 and 31 December 2014 were eligible. These dates were chosen in previous analyses to explore health inequalities in preschool wheeze [26]. Exclusion criteria were children with chronic respiratory conditions other than asthma.

Acute wheeze episodes were defined using diagnostic codesor recorded visits to emergency care or admitted patient care for asthma/wheeze. The code lists used are available on GitHub *via* https://github.com/dkhl1/ip2am; code list creation details are available *via* https://github.com/NHLI-Respiratory-Epi/SNOMED-CT-codelists.

The validation cohort comprised children aged 1–5 years with linked data in the SAIL Datalink [18–20]. Children ≤5 years with at least one acute wheeze either between 1 January 2017 and 31 December 2018 or between 1 January 2020 and 31 December 2020 were eligible. The same exclusion criteria were applied, and code lists for acute wheeze episodes were converted to read codes.

A sample size calculation indicated that a minimum prevalence of 5%, maximum number of model parameters of 30 and sample size of 35 000 would result in a minimum observed C-statistic of 0.61 [27].

### Outcome and predictors

The first acute wheeze recorded within the relevant timeframe was defined as a child's index episode. The primary outcome was a subsequent acute wheeze (presenting to any healthcare setting) within 12 months of their index episode, with a secondary outcome of a hospitalisation from an acute wheeze episode.

Candidate predictors in the development cohort, identified through previous research [16], included demographics, clinical and treatment history and additional diagnoses. Specifically, the following predictors were considered: age; sex; ethnicity (according to modal recorded HES ethnicity [28]); socioeconomic status (Index of Multiple Deprivation [29]); practice location; rural–urban classification [30]; number of wheeze episodes ever and if any occurred in 12 months prior to index; healthcare setting of the index episode (primary care or hospitalisation); any previous hospital admissions with wheeze; number of SABA reliever prescriptions in 12 months prior to index date; number of ICS prescriptions in 12 months prior to index date; atopy; bronchiolitis; prematurity at birth; and gastro-oesophageal reflux disease. The number of wheeze episodes ever prior to index date, and the number of SABA and ICS prescriptions in the 12 months prior to index, were categorised into 0, 1–2, 3–4 or 5+ for ease of implementation. No form of clustering beyond practice location was considered.

### Model development and validation

A risk prediction model for subsequent episodes of wheeze was developed using covariates informed by clinical and statistical relevance. Multivariable logistic regression was used to model the primary outcome of the risk of one or more further wheeze episodes within 12 months of an index episode. The Bayes information criterion identified which predictors were most statistically relevant while preventing overfitting [31]. The final model contained a parsimonious combination of the most statistically and clinically significant variables [32]. The identified predictors were previously found to not be significantly multicollinear in these data [16].

The model was developed on the full dataset using a complete case analysis, as minimal missing data were anticipated and to simplify model reproduction. It was internally validated through 1000 bootstrap samples [33], evaluating calibration (calibration-in-the-large (CITL), calibration slope, calibration plot) and discrimination (concordance C-statistic [7]) [34]. The prediction model was externally validated using the same performance metrics.

Assumptions made in the model development were varied in a sensitivity analysis to assess their impact on performance statistics, including not discretising/considering alternative functional forms of continuous predictors and including all potential predictors.

Potential risk groups were explored to categorise children into low, medium or high-risk categories. Sensitivity, specificity, positive predictive value (PPV) and negative predictive value (NPV) were assessed across a range of thresholds for risk groups (defined by individual predicted risk scores).

The final model will be implemented in the future as a user-friendly web tool developed in consultation with patient and clinical stakeholders. The tool will be freely available online for clinicians and the public, and allow users to obtain a prediction of a subsequent wheeze episode in the following 12 months. A simplified model will also be available with rounded regression coefficients for clinical usability where web access is unavailable. This will be developed with input from patients and clinicians to ensure maximal uptake and simplicity of design, such as colour-coding risk groups. An example of how the web tool will appear is available on GitHub *via* https://github.com/dkhl1/ip2am (not yet ready for clinical use).

Statistical analyses were conducted in STATA versions 18/19. Applications to CPRD can be made at https://www.erap.cprd.com/.

## Results

### Characteristics

The full demographics of the development cohort are previously documented [16]. Table 1 presents baseline characteristics for the development and validation cohorts.

**TABLE 1:** Baseline characteristics of the development and validation cohorts

Variable	Development cohort 2013–2014^#^	Validation cohort A 2017–2018^¶^	Validation cohort B 2020^¶^
**Whole cohort**	42 820 (100)	7712 (100)	1991 (100)
**Subsequent acute wheeze (within 12 months of index)**			
Yes (primary outcome)	16 962 (39.6)	2899 (37.6)	745 (37.4)
**Subsequent hospitalisation within 12 months of index)**			
Yes (secondary outcome)	7443 (17.4)	1527 (19.8)	407 (20.4)
**Sex**			
Female	15 791 (36.9)	2807 (36.4)	716 (36.0)
Male	27 029 (63.1)	4905 (63.6)	1275 (64.0)
**Recorded ethnicity**			
White	31 433 (73.4)	-	-
South Asian	4838 (11.3)	-	-
Black	2446 (5.7)	-	-
Other	1102 (2.6)	-	-
Mixed	1922 (4.5)	-	-
Missing/unknown	1079 (2.5)	-	-
**Atopy, Yes**	21 445 (50.1)	5066 (65.7)	750 (37.7)
**Bronchiolitis, Yes**	9141 (21.4)	1761 (22.8)	574 (28.8)
**Age at index, years**			
0	10 597 (24.8)	2214 (27.5)	371 (18.6)
1	13 282 (31.0)	2181 (28.3)	523 (26.3)
2	8190 (19.1)	1443 (18.7)	412 (20.7)
3	6326 (14.8)	1127 (14.6)	392 (19.7)
4	3686 (8.6)	837 (10.9)	293 (14.7)
5	739 (1.7)	0 (0.0)	0 (0.0)
**SABA prescriptions (12 months prior to index)**			
0	27 198 (63.5)	4878 (63.3)	1118 (56.2)
1–2	11 403 (26.6)	1947 (25.3)	524 (26.3)
3–4	2671 (6.2)	492 (6.4)	168 (8.4)
5+	1548 (3.6)	395 (5.1)	181 (9.1)
**ICS prescriptions (12 months prior to index)**			
0	37 825 (88.3)	6801 (88.2)	1606 (80.7)
1–2	3034 (7.1)	459 (6.0)	171 (8.6)
3–4	1189 (2.8)	208 (2.7)	89 (4.5)
5+	772 (1.8)	244 (3.2)	125 (6.3)
**Healthcare setting of index episode**			
Primary care or emergency department	29 967 (70.0)	7516 (97.5)	1916 (96.2)
Hospitalisation	12 853 (30.0)	196 (2.5)	75 (3.8)
**Previous attack (12 months prior to index)**	4358 (10.2)	1613 (20.9)	728 (36.6)
**Previous hospitalisation** (**12 months prior to index)**	2163 (5.1)	1307 (17.0)	536 (26.9)
**Number of previous attacks (ever prior to index)**			
0	33 779 (78.9)	5317 (68.9)	1019 (51.2)
1–2	5776 (13.5)	1704 (22.1)	613 (30.8)
3–4	1712 (4.0)	462 (6.0)	196 (9.8)
5+	1553 (3.6)	229 (3.0)	163 (8.2)
**Number of previous hospitalisations (ever prior to index)**			
0	27 171 (63.5)	5885 (76.3)	1250 (62.8)
1–2	14 329 (33.5)	1495 (19.4)	543 (27.3)
3–4	927 (2.2)	228 (3.0)	125 (6.3)
5+	393 (0.9)	104 (1.4)	73 (3.7)

Data are presented as n (%). SABA: short acting β_2_-agonist; ICS: inhaled corticosteroids. ^#^: Clinical Practice Research Datalink (CPRD) cohort; ^¶^: Secure Anonymised Information Linkage (SAIL) cohort.

The development cohort consisted of 42 820 children with a history of wheeze, of whom 16 962 (39.6%) experienced at least one subsequent wheeze and 7443 (17.4%) were hospitalised within 12 months of their index episode. 40 826 children (95.3%) had no missing covariate information with data available for the complete case analysis. Of the predictors included in the final model, only ethnicity had missing data (2.5%).

The two SAIL validation cohorts had similar baseline characteristics, and were broadly similar to the CPRD development cohort with some notable differences. The validation cohorts were smaller (as expected due to the smaller population of SAIL); the 2020 validation cohort was smaller than the 2017–2018 cohort because it covered a shorter time period and due to decreased healthcare contact during the COVID-19 pandemic. In the first/second validation cohorts, respectively, 2899 (37.6%) and 745 (37.4%) experienced at least one subsequent wheeze episode, while 1527 (19.8%) and 407 (20.4%) were hospitalised within 12 months of their index episode A similar split of boys and girls was observed, alongside a similar distribution of ages at index episode. The largest difference was in the healthcare setting of the index episodes; in the development cohort, 70% of index episodes were observed in primary care or emergency departments; in both validation cohorts, this was >95% of episodes. Ethnicity could not be included in the validation cohorts, as due to the lower cohort sizes combined with the high proportion (>90%) of White ethnicity in Wales [35], there were too small frequencies among non-White groups, rather than due to missingness.

### Selection of model predictors

The following predictors minimised the Bayes information criterion: healthcare setting of index episode; history of wheeze in the 12 months prior to index; diagnosis of bronchiolitis; diagnosis of atopy; age at index; sex; number of wheeze episodes ever prior to index episode; number of hospitalisations ever prior to index episode; and number of SABA prescriptions in 12 months prior to index. Ethnicity and the number of ICS prescriptions in 12 months prior to index were also included as these indicated strong, clinically plausible predictive relationships with the primary outcome. The number of ICS prescriptions indicated a positive relationship with the outcome; the more ICS prescriptions in the 12 months prior to index, the lower the risk of a subsequent wheeze.

### Model estimates and performance

Model estimates of adjusted odds ratios (aORs) and confidence intervals (CIs) from the final logistic regression model for the primary outcome are shown in figure 1.

**FIGURE 1:**
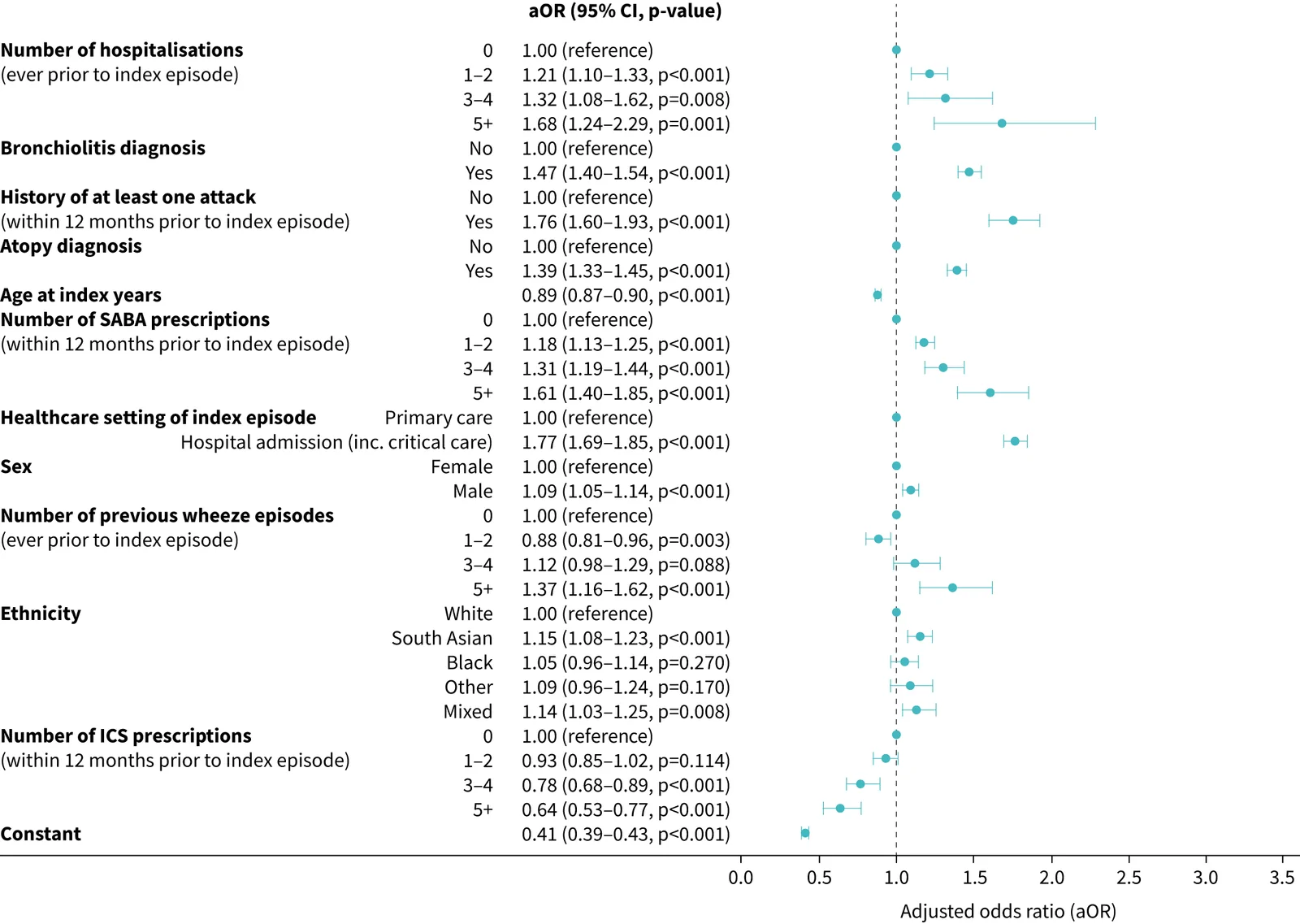
Forest plot of adjusted odds ratios from final logistic regression model for subsequent wheeze attack. SABA: short acting β_2_-agonist; ICS: inhaled corticosteroids.

The healthcare setting of the index episode was the strongest predictor of a subsequent wheeze episode, with a child presenting to a hospital having nearly double the odds of a subsequent wheeze (aOR=1.77, 95% CI 1.69–1.85) than a child presenting to primary care or emergency department. History of attacks within the year prior to the index episode also strongly increased the odds of a subsequent attack (aOR=1.76, 95% CI 1.60–1.93). White children were at the lowest risk of a subsequent wheeze episode across ethnicities, and South Asian children were estimated to be at the highest risk. The older a child was at index, the lower their odds of a subsequent wheeze (aOR=0.89, 95% CI 0.87–0.90). A higher number of ICS prescriptions also decreased the odds of a subsequent wheeze.

The calibration plot in the development cohort for the final model for any subsequent wheeze episode is shown in figure 2, produced using *pmcalplot* [34].

**FIGURE 2:**
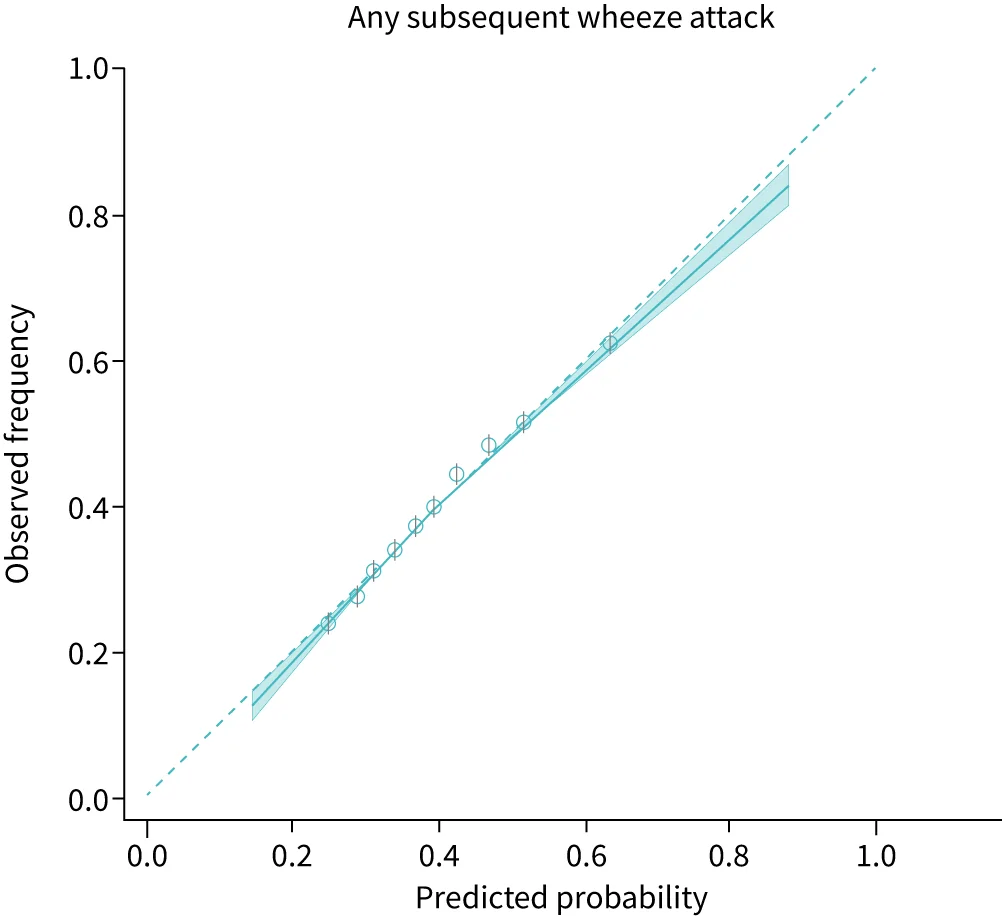
Calibration plot from final logistic regression model for subsequent wheeze episode.

The plot indicates good calibration, with close agreement to the 45-degree line and a narrow CI. The groups were fairly well dispersed, with some minor indication that the model overpredicted the risk of a subsequent wheeze among those at extremes of predicted risk. Equivalent plots to figures 1 and 2 for wheeze hospitalisation are available in supplementary figures S1 and S2.

### Model validation

Table 2 shows estimates of the calibration slope, calibration in the large and C-statistic in the development cohort, from the bootstrap samples for internal validation and from the external validation cohort, for both models.

**TABLE 2:** Calibration slope, calibration-in-the-large (CITL) and C-statistic from the final model and internal validation *via* bootstrap samples for any wheeze episode (primary outcome) and wheeze hospitalisation (secondary outcome)

Cohort	Calibration slope	CITL	C-statistic
**Subsequent wheeze episode**			
Final model	1.000	0.000	0.634
Bootstrap 95% CI	(0.959–1.043)	(−0.020–0.0020)	(0.629–0.639)
External cohort A	0.944	0.035	0.625
External cohort B	0.859	−0.143	0.638
**Wheeze hospitalisation**			
Final model	1.000	0.000	0.678
Bootstrap 95% CI	(0.959–1.040)	(−0.026–0.025)	(0.671–0.685)
External cohort A	0.908	−1.785	0.631
External cohort B	0.721	−1.855	0.615

The performance measures indicated excellent calibration. The calibration slope measures calibration across different risk groups, while the CITL is a measure of absolute predictions of risk (*i.e.* the constant term in figure 1). The C-statistic of 0.634 (95% CI 0.629–0.639) suggests moderate model discrimination, which could be improved either through the inclusion of further predictors or alternative functional forms of current predictors. However, none of the sensitivity analyses yielded a C-statistic of >0.636. The secondary model for wheeze hospitalisation was also very well calibrated and showed slightly better discrimination (C-statistic=0.678, 95% CI 0.671–0.685).

The external validation underscored the strong calibration and moderate discrimination for the primary model predicting a subsequent wheeze episode, particularly in the earlier cohort (index episode in 2017–2018). The CITL from the later external validation cohort was −0.143, suggesting a small amount of overprediction of overall risk in this cohort. Model performance in the external cohorts for the secondary model was poorer, with reasonable calibration across risk groups (calibration slope of 0.908 and 0.721) but poor prediction of absolute risk (CITL of −1.785 and −1.855). This suggests that the primary model is able to generalise well to wider populations, while the secondary model may require a wider range of predictors or recalibration of predictor effects to be used beyond the English population. Performance was also generally poorer in the more recent external cohort, where the sample size was smaller and predictor distribution more varied from the development cohort.

The sensitivity, specificity, PPV and NPV across different risk score thresholds that the model may predict (in the development cohort) are plotted in figure 3. Specific values are shown for categorising children into low risk, medium risk and high risk based on predicted risk thresholds of 30% and 50%.

**FIGURE 3:**
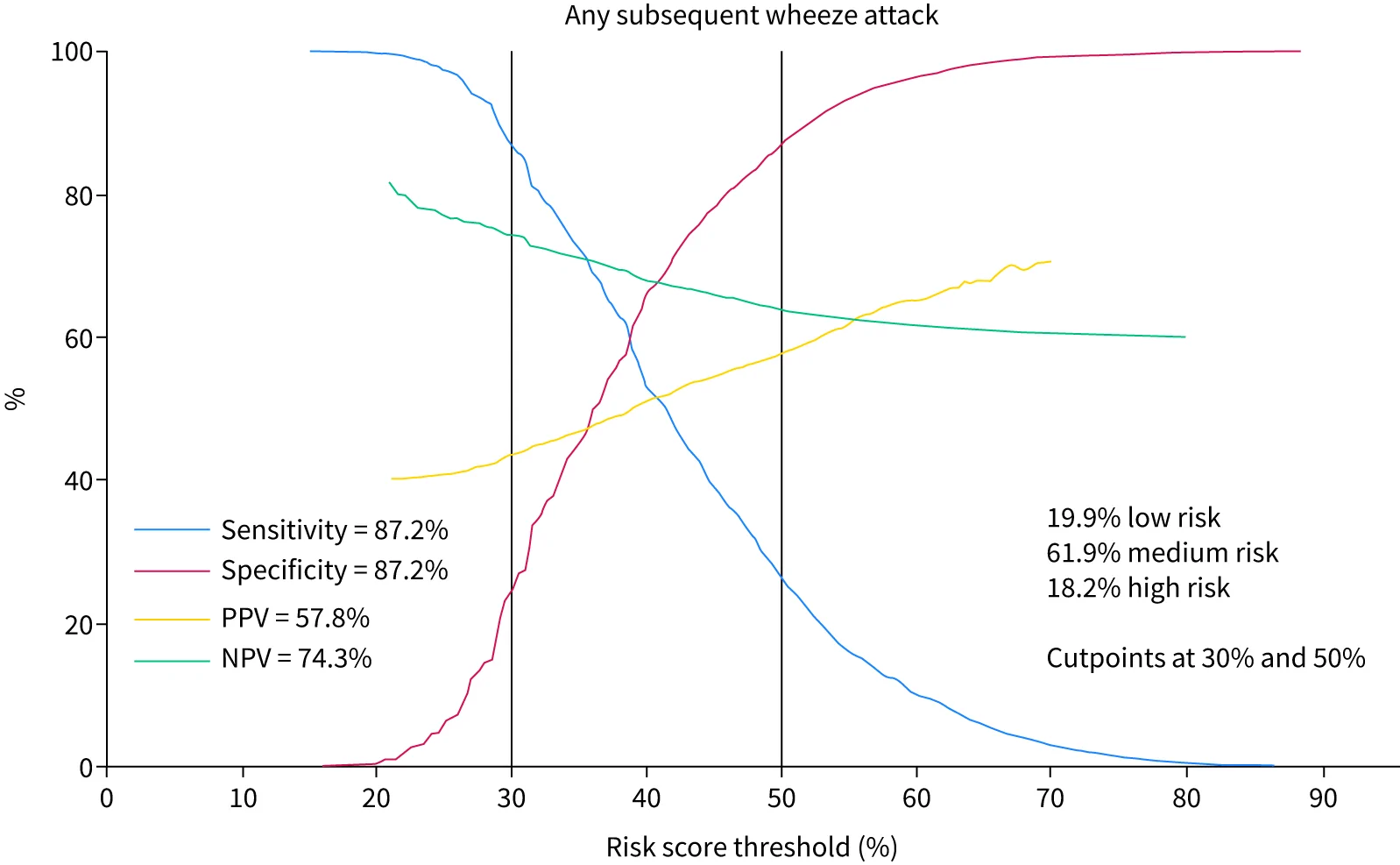
Sensitivity, specificity, positive predictive value (PPV) and negative predictive value (NPV) across different risk score thresholds.

The categorisation shown in figure 3 assign 19.9% of children to the low-risk group, 61.9% to the medium-risk and 18.2% to the high risk group. Predicting that low-risk children will not experience a subsequent wheeze attack in the next 12 months, high-risk children will experience a subsequent attack and medium-risk children may or may not and therefore require closer monitoring results in a child in the high-risk group having at least a 57.8% chance of an attack (PPV) and a child in the low-risk group having at least a 74.3% chance of not having such an attack (NPV). Different thresholds can prioritise different performance metrics and result in a different split of children across risk groups; for instance, thresholds at 32.0% and 47.5% assigns roughly a quarter of children to the low-risk and a further quarter to the high-risk groups, with minimal impact on PPV and NPV and a slight reduction in sensitivity and specificity (to 80.5% and 82.9%, respectively). Supplementary table S1 presents metrics for a wide variety of different thresholds to highlight this.

## Discussion

This study presents the development and external validation of a prognostic model to find preschool children at risk of experiencing a subsequent wheeze episode or hospitalisation within 12 months of an acute wheeze presentation. Using large, representative datasets from England (42 820 children) and Wales (9703 children across two cohorts), the model demonstrated good calibration and moderate discrimination, offering a potentially valuable tool for early risk stratification in clinical practice.

The strongest predictors of a subsequent wheeze episode included the healthcare setting of the index episode, with hospital presentations nearly doubling the odds compared with primary care. Other key predictors were a history of wheeze in the preceding year, diagnoses of bronchiolitis and atopy, younger age, male sex and higher SABA use. Notably, increased ICS prescriptions in the prior year were associated with reduced risk, consistent with the potential protective effect of regular preventer therapy [7, 8]. This finding should be interpreted with caution as it may reflect confounding by indication and treatment adherence. While the model suggests a possible protective effect, clinicians may be more likely to prescribe ICS to children with more severe disease, which could underestimate the benefit of treatment in routine data. Additionally, as our model relies on prescription records, we cannot account for actual medication usage (adherence) or inhaler technique, which influence outcomes in clinical practice. However, these findings align with existing literature for both children and adults and reinforce the clinical relevance of these variables in assessing future risk [16, 36–38].

The model's performance was robust across internal and external validation, with calibration slope and calibration-in-the-large values close to ideal (1 and 0, respectively). Discrimination, as measured by the C-statistic, was moderate (0.634 for subsequent wheeze and 0.678 for hospitalisation), suggesting the model can reasonably differentiate between high- and low-risk children, though there remains room for improvement. While the discrimination limits the model's ability to precisely distinguish individuals at the extremes of risk, such levels of discrimination are not uncommon when using routinely collected data [39, 40]. Importantly, the strong calibration is essential for clinical applicability when models are intended to support risk stratification. In this context, the model is likely to be most useful for identifying subgroups of children at relatively higher risk (PPV 57.8%) who may benefit from closer monitoring or early intervention, or lower-risk groups (NPV 74.3%) where reassurance and safety-netting are appropriate. Sensitivity analyses did not yield substantial gains in discrimination, indicating that the current predictor set may be approaching the limits of available routine data. The model performed well under external validation, while the secondary model predicting subsequent hospitalisations was more poorly calibrated in the external cohorts, suggesting further predictor ascertainment may be required prior to clinical implementation.

Clinically, this model could support decision-making by identifying children who may benefit from closer monitoring or early initiation of preventative therapies such as ICS. It may also help guide referrals and resource allocation. The sensitivity, specificity, PPV and NPV presented across different thresholds in figure 3 can inform how potentially at-risk children may be identified, and how different categories might be defined or adapted to clinical priorities.

However, some limitations are acknowledged. Differences in coding practices and healthcare delivery between the CPRD and SAIL datasets may have influenced predictor and outcome ascertainment. The development cohort included a higher proportion of hospital-based index episodes (30%) compared to the validation cohorts (<4%); and the lack of ethnicity data in the validation cohorts limited our ability to externally validate the risk for non-White ethnic groups. However, our primary model maintained strong calibration despite these geographical and demographic variations, suggesting the core clinical predictors are robust across different UK healthcare settings. The second model (predicting hospitalisations) was less well calibrated-in-the-large in the external cohorts, suggesting this model may be more dependent on regional variations in hospital admission criteria and pathways. The timelines of the two cohorts provide the potential for further differences, although we would not expect risk factors to have changed between the two.

Additionally, the model relies on routinely collected data, which may omit relevant clinical nuances such as symptom severity or environmental exposures. Future research could explore the integration of additional predictors, including biomarkers such as eosinophil count (although these may be less readily available for preschool children, particularly those without a previous hospital admission). The secondary model aimed at predicting hospitalisations was poorly calibrated in the large external cohorts, suggesting this model requires further refinement.

This risk prediction model offers a promising approach to identifying preschool children at risk of recurrent wheeze or hospitalisation. This aligns with national and international guidelines that emphasise the importance of identifying children at risk of future wheeze attacks as a critical component of asthma care, to enable timely intervention and reduce avoidable hospitalisation [10, 11]. Its strong calibration and future implementation as a web-based tool support its potential utility in clinical practice.

## Data Availability

Data sets used in this analysis were obtained *via* a CPRD institutional licence. Requests for data should be made directly to the CPRD *via* their online application portal (https://cprd.com/researchapplications). Code lists used to identify the cohort are available at https://github.com/dkhl1/ip2am. This study makes use of anonymised data held in the Secure Anonymised Information Linkage (SAIL) Databank. We would like to acknowledge all the data providers who make anonymised data available for research. SAIL is not responsible for the interpretation or further analysis of the study findings. The final model will be implemented in the future as a user-friendly web tool and will be freely available online for researchers, clinicians and the public.

## Supplementary material

**Please note:** supplementary material is not edited by the Editorial Office, and is uploaded as it has been supplied by the author. Supplementary material 00337-2026.SUPPLEMENT
