# Supplementary material for: Development and external validation of a risk prediction tool for recurrent wheeze attacks in preschool children in England
Source: ERJ Open Res. 2026 Oct 19;12(5):00337-2026. doi: 10.1183/23120541.00337-2026 (PMC13678849; doi:10.1183/23120541.00337-2026)
Supplement: Supplementary file 1 [file 00337-2026.SUPPLEMENT.pdf]

Supplementary Materials

The model selection for the secondary outcome, a wheeze hospitalisation, identified the same predictors with the addition of ethnicity as minimising the Bayes information criterion, as well as a history of hospitalisation (rather than any wheeze episode) in the 12 months prior to index. The number of ICS prescriptions was again clinically significant.

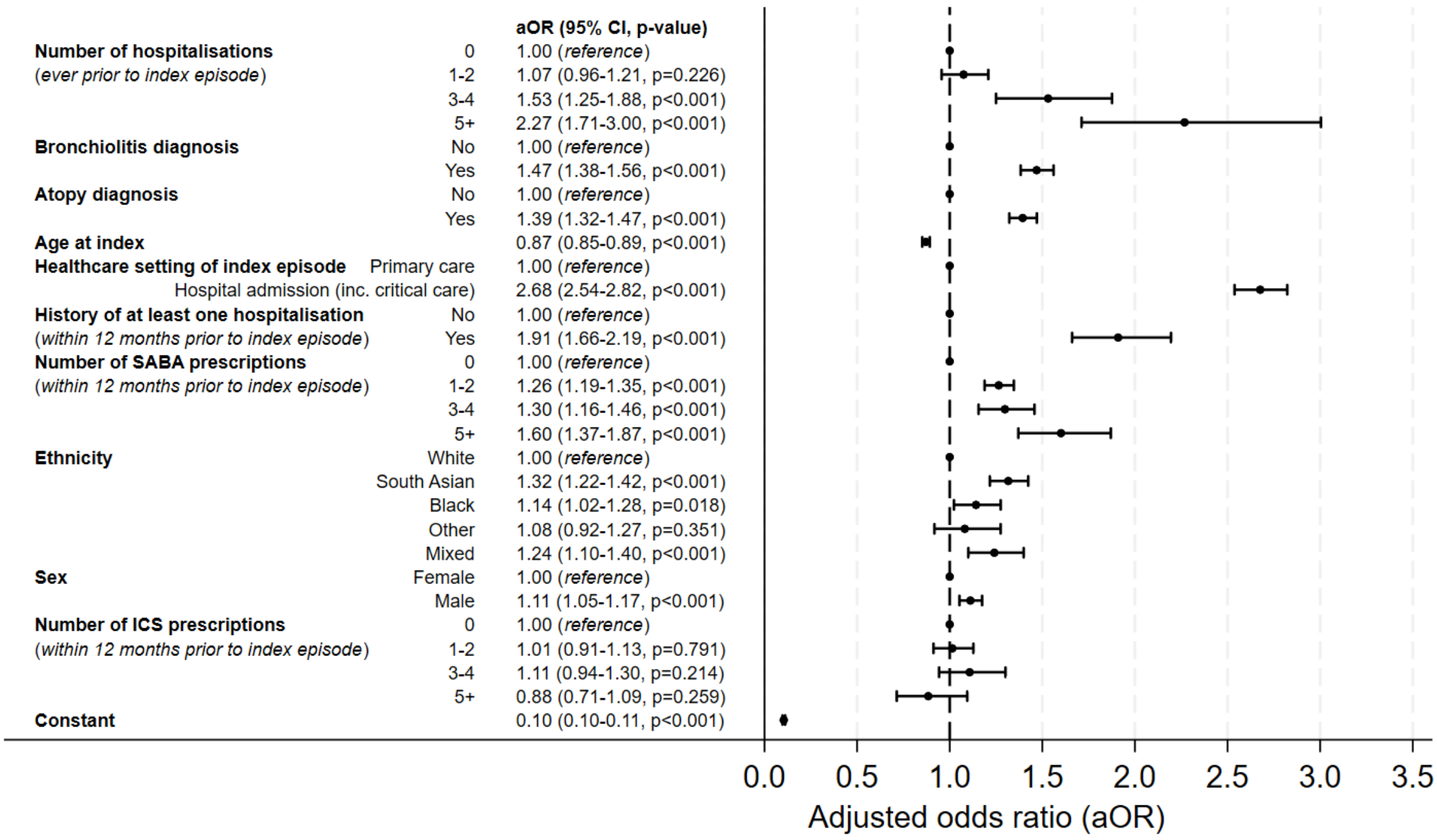

Figure S1: Forest plot of adjusted odds ratios from final logistic regression model for hospitalisation. SABA = short acting beta-2 agonist. ICS = inhaled corticosteroids.

The constant term indicates the odds of the outcome in the 12 months after index for an individual with all predictors set to the reference level; thus, absolute odds of the outcome can be calculated for a particular set of predictor values by multiplying the constant by the relevant aORs, and absolute risk of the outcome calculated by absolute odds/(1 + absolute odds).

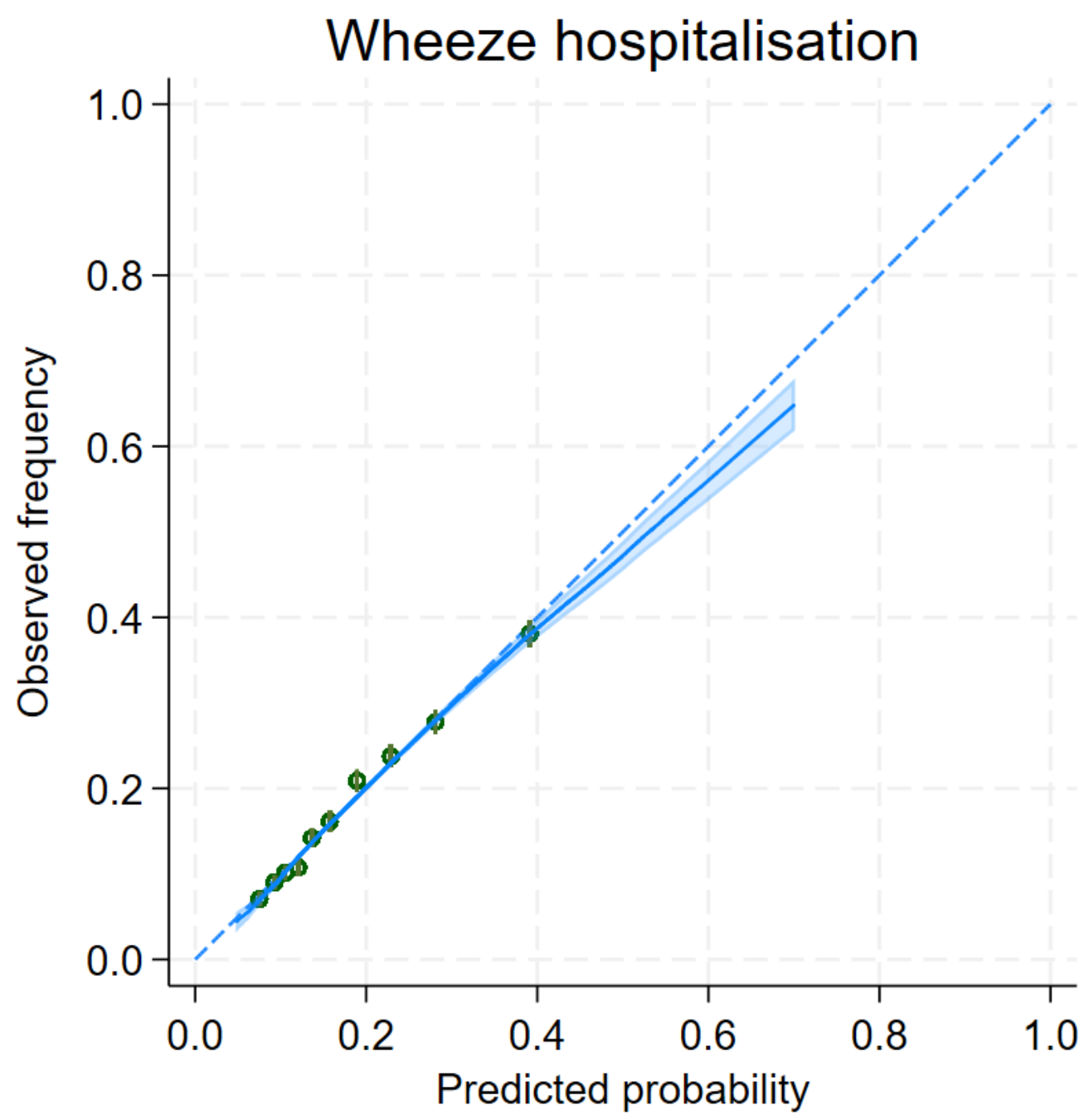

Figure S2: Calibration plot from final logistic regression model for hospitalisation.

The healthcare setting of the index episode and diagnoses of bronchiolitis or atopy were also strong predictors of a hospitalisation within the following 12 months, as were a history of another wheeze episode and the number of SABA prescriptions (both in the last year). Older children were also less at risk, as were girls; a 5-year-old girl was estimated to have half the odds of a hospitalisation than a 1-year-old boy. The model appeared well calibrated across risk groups, with a small amount of risk overprediction in children most at risk.

Model performance in the external cohorts for the secondary model was poorer, with only moderate preservation of relative risk ranking (calibration slopes of 0.908 and 0.721) but substantial miscalibration of absolute risk estimates, as reflected by strongly negative calibration in the large values (−1.785 and −1.855). These findings indicate that the secondary model systematically overpredicted hospitalisation risk in both external cohorts, likely reflecting differences in baseline event rates and predictor distributions between the development and validation cohorts, such as the healthcare setting of index episode. While the secondary model retained some ability to distinguish higher from lower risk children, the degree of overprediction limits applicability in external settings without recalibration.

Table S1: Sensitivity, specificity, negative predicted value (NPV), positive predicted value (PPV) and proportion of children in each risk group for subsequent wheeze attack across a range of thresholds.

Lower Threshold	Upper Threshold	Low Risk	Medium Risk	High Risk	Sensitivity	Specificity	PPV	NPV
25	30	4.8	15.1	80.1	97.2	24.7	43.5	77.0
25	35	4.8	33.6	61.6	97.2	45.6	47.0	77.0
25	40	4.8	53.9	41.3	97.2	66.4	51.2	77.0
25	45	4.8	66.3	28.8	97.2	78.2	54.5	77.0
25	50	4.8	77.0	18.2	97.2	87.2	57.8	77.0
25	55	4.8	84.8	10.4	97.2	93.4	61.9	77.0
30	35	19.9	18.5	61.6	87.2	45.6	47.0	74.3
30	40	19.9	38.8	41.3	87.2	66.4	51.2	74.3
30	45	19.9	51.2	28.8	87.2	78.2	54.5	74.3
30	50	19.9	61.9	18.2	87.2	87.2	57.8	74.3
30	55	19.9	69.7	10.4	87.2	93.4	61.9	74.3
35	40	38.5	20.3	41.3	72.3	66.4	51.2	71.2
35	45	38.5	32.7	28.8	72.3	78.2	54.5	71.2
35	50	38.5	43.4	18.2	72.3	87.2	57.8	71.2
35	55	38.5	51.2	10.4	72.3	93.4	61.9	71.2
40	45	58.7	12.5	28.8	52.9	78.2	54.5	67.9
40	50	58.7	23.1	18.2	52.9	87.2	57.8	67.9
40	55	58.7	30.9	10.4	52.9	93.4	61.9	67.9
45	50	71.2	10.7	18.2	39.3	87.2	57.8	65.9
45	55	71.2	18.4	10.4	39.3	93.4	61.9	65.9
50	55	81.8	7.8	10.4	26.3	93.4	61.9	64.0
